# Integration of functional genomics data to uncover cell type-specific pathways affected in Parkinson's disease

**DOI:** 10.1042/BST20210128

**Published:** 2021-09-28

**Authors:** Viola Volpato

**Affiliations:** UK Dementia Research Institute, Cardiff University, Cardiff CF24 4HQ, U.K.

**Keywords:** computational biology, data integration, Parkinsons disease, single cells

## Abstract

Parkinson's disease (PD) is the second most prevalent late-onset neurodegenerative disorder worldwide after Alzheimer's disease for which available drugs only deliver temporary symptomatic relief. Loss of dopaminergic neurons (DaNs) in the substantia nigra and intracellular alpha-synuclein inclusions are the main hallmarks of the disease but the events that cause this degeneration remain uncertain. Despite cell types other than DaNs such as astrocytes, microglia and oligodendrocytes have been recently associated with the pathogenesis of PD, we still lack an in-depth characterisation of PD-affected brain regions at cell-type resolution that could help our understanding of the disease mechanisms. Nevertheless, publicly available large-scale brain-specific genomic, transcriptomic and epigenomic datasets can be further exploited to extract different layers of cell type-specific biological information for the reconstruction of cell type-specific transcriptional regulatory networks. By intersecting disease risk variants within the networks, it may be possible to study the functional role of these risk variants and their combined effects at cell type- and pathway levels, that, in turn, can facilitate the identification of key regulators involved in disease progression, which are often potential therapeutic targets.

## Introduction

Currently, 2–3% of the population over 65 years of age are living with Parkinson's disease (PD), making this disorder the most prevalent late-onset neurodegenerative disorder worldwide after Alzheimer's disease (AD) [1]. Dopaminergic neurons (DaNs) in the substantia nigra pars compacta (SNpc) of PD patients present distinctive neuronal inclusions of alpha-synuclein, or Lewy bodies [1,2] and their preferential loss gives rise to specific motor features such as tremor at rest, muscle rigidity and bradykinesia [3]. For this, the primary focus of PD research to date has been on DaNs that, due to their unique cellular features, are also the most vulnerable cell type in the disease compared with other neuronal populations in the brain; complex morphology with extensive and unmyelinated axonal innervation conveys, indeed, an exceptionally high energy cost [4] and, high calcium flux and dopamine metabolism [5] make them particularly susceptible to various stressors [6].

Several large consortia have now assembled genome-wide association studies (GWAS) that, by associating genetic loci with clinical diagnoses of PD [7–9] or with PD characteristic pathological features [10], have led to the identification of more than 20 causal genes from familial PD cases and over 90 risk loci contributing to 16–36% of PD heritability [8,11,12]. Recent advances in induced pluripotent stem cell (iPSCs) disease models and development of powerful omics and computational tools are enabling genome-wide characterisation on how these identified mutations alter context-specific biological processes. This is also contributing to growing evidence that cell types other than DaNs, such as astrocytes [13], microglia [13,14] and oligodendrocytes (ODCs) [15,16] are involved in PD pathogenesis. As GWASs have grown in size and power, so has the quality and scope of functional information that can be used to annotate the genome [17,18], providing new opportunities to further address the cellular specificity of disease heritability [3].

In this review, I first present recent findings on PD-affected cell type-specific pathways identified through current technologies including analysis of iPSC-derived disease models and intersection of disease genetic risks with brain transcriptomic atlases. I then describe cell type-specific functional genomic annotations and more recent methodologies for their integrative analysis that can provide stronger evidence for their association to the disease and can help elucidate the cascade of molecular events contributing to PD mechanisms at cell-type resolution.

## Cell type-specific pathways affected in PD

The recent development of iPSC technology resulted in a profound improvement of disease-modelling strategies for neuroscience research enabling the analysis of phenotypic abnormalities, intracellular pathways relevant for disease and cell response under stress challenges [19]. For PD, this translated in a better understanding of the role and phenotypic consequences of PD familial mutations in models of DaNs, astrocytes and microglia. SNCA-A53T mutation has been proposed to affect DaN-specific processes such as endoplasmic reticulum (ER), protein trafficking and ER stress [20], mitochondrial function [21], axonal degeneration, neurite growth [22], and cellular bioenergetics [23]. LRRK2-G2019S mutation has shown DaN-specific effect at the level of mitochondria [24], but also a reduced neuroprotective capacity in astrocytes [25] and microglia-specific modulation of cytokine production and glycolytic switch in response to IFN-γ resulting in neurotoxicity [26]. GBA-N370S mutation exhibited increased ER stress, autophagic and lysosomal perturbations, and elevated α-synuclein release in DaNs [27]. Furthermore, Parkin and PINK1 loss-of-function mutations associated with early-onset familial PD showed dysregulation of mitochondrial quality control [28,29], that can, in turn, activate the NLRP3 inflammasome both in macrophages in a Parkin-dependant manner [30] and in astrocytes [31]. Single cell RNA-seq (scRNA-seq) experiments, that measure the distribution of gene expression levels in single cells, have further improved over bulk RNA-seq approaches which failed to dissect cell type-specific contributions to disease pathology [3,32]. For instance, deep-coverage scRNA-seq of iPSC-derived DaNs highlighted GBA-N370S mutation effects in neuronal function, microtubule function and formation, neurite and axonal outgrowth, protein secretion and trafficking, and ER stress [33], while low-coverage scRNA-seq of iPSC-derived DaNs revealed a subpopulation of DaNs showing higher vulnerability to PD-like rotenone-induced stress and to SNCA-A53T mutation with dysregulation of autophagy and ubiquitin-, heat shock- and oxidative stress response-related genes [34]. Only recently, single cell transcriptomic atlases of mouse [16,35,36] and human post-mortem brain regions [15,37,38] have shed new light on the causal effect of the genetic risks of brain-related diseases at cell type resolution [39]. PD-related works on mouse [16] and human [15] brain tissues have confirmed convergence of causal genetic risk effects in different neuronal populations, such as cholinergic neurons, enteric neurons and SNpc DaNs, as well as in ODCs. At the pathway level, PD genetic risk mainly affects previously reported DaN-specific processes such as mitochondrial functioning, protein ubiquitination, endocytosis and oxidative phosphorylation, and novel ODC-specific pathways related to protein phosphorylation and regulation of gene expression and of metabolic processes [15].

**Figure 1.**
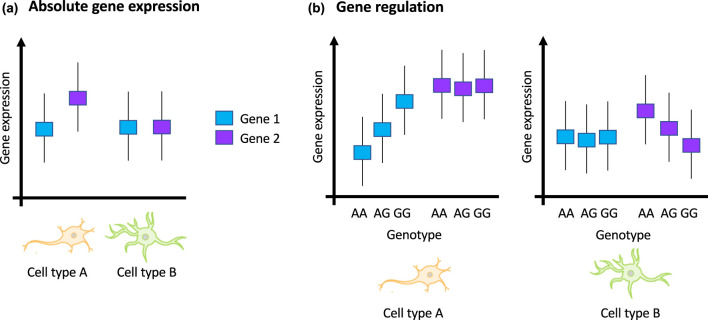
Cell type gene specificity defined by gene regulation. (**a**) based on current methodologies that rely on absolute gene expression levels, Gene 1 is not defined as cell type-specific while Gene 2 is defined as cell type A specific showing higher gene expression in cell type A than in cell type B. (**b**) based on methods that look at variation in gene regulation mechanisms, such as expression quantitative trait loci or eQTLs, across cell types, Gene 1 is defined as cell type A specific while Gene 2 is defined as cell type B specific. These genetic effects on gene regulation can be more informative in deciphering the functional role of disease-associated variants at cell type resolution.

Despite these advances, we are still far from having a complete knowledge on the functional mechanisms by which risk variants mediate disease susceptibility at cell type level. It is likely that instead of the absolute level of gene expression, gene expression change, i.e. its regulation, between cell types is key to define cell type gene specificity (Figure 1). Thus, different sets of genes and, consequently, a wider range of cellular processes have yet to be revealed and analysed in terms of how they interact with one another and between different cell types in healthy and PD brains. Such analyses may help shed light on the underlying biology of DaN-specific vulnerability and on the dysfunction of other cell types that, in turn, will contribute to identifying potential novel drug targets. In light of this, in the next section I will describe current functional omics data as better reference to define cell type gene specificity, and recent methodologies for integrated analysis of the effects of disease-associated variants on gene expression, gene regulation and gene interactome at cell-type resolution.

## How to obtain and integrate cell type-specific gene functional annotations

### Cell type specificity of gene regulatory mechanisms

The progressive increase in the number of publicly available brain cell-type and tissue-specific annotations, including those released by the UK Brain Expression Consortium [40] and the Genotype-Tissue Expression (GTEx) [41], the Roadmap Epigenomics Project [42] and the PsychEncode Project [43], as well as their increasing sample size, sequencing depth, and tissue/cell type resolution can improve our power to interpret GWAS results in the context of regulatory effects [44,45]. Disease-associated variants have, indeed, shown strong enrichment with functional genomic annotations for gene expression regulation mechanisms, such as DNase I hypersensitive sites, mRNA-transcription factor interactions, histone modifications, and expression and splicing quantitative trait loci (eQTLs and sQTLs, respectively) [46–48]. Furthermore, not only these regulatory mechanisms have an important role in neurodegeneration [46], but also they are often highly cell type-specific [49]. A recent study, that expanded the total number of putative brain enhancers by 87%, has shown that cell type specificity is mainly captured within the enhancer repertoire with active histone (enhancer) marks H3K4me1 and H3K27ac better at resolving cell type classification than promoter marks [49]. Additionally, a study on DNA methylation between controls and schizophrenia patients has revealed that the methylomes of the two cell types under investigation were highly distinct and that disease-associated differential DNA methylations tended to occur in cell type differentially methylated sites, highlighting the significance of cell type-specific epigenetic dysregulation in a complex brain-related disorder [50]. To translate from genetic signals to mechanisms, associations with eQTLs have also shown great potential. In particular, *cis*-eQTLs can aid GWAS interpretation by measuring direct effects of local genetic variants and identifying direct links between genes and phenotypes, whereas *trans*-eQTLs, that measure indirect effects of distal variants, can identify downstream genes and pathways on which the effects of disease variants converge [51]. Notably, eQTLs have long been suggested to exert their influence in a cell-specific manner, and the large portion of unresolved eQTLs may be attributable to the cell type dependent effects of these eQTLs [52]. Cell type-specific eQTL studies may also reduce the detection of false-positive variants associated with disease that have often emerged as potential limitations of bulk RNA-seq-based eQTL research. By using a large number of cells, scRNA-seq experiments may significantly reduce the number of samples required for eQTL detection [52], from the suggested ∼80 in bulk analysis [41] to less than 40 for single cell analysis [53,54]. Furthermore, contribution of alternative splicing events to the molecular diversity across brain regions and cell types is just starting to emerge. Although some studies have suggested that the effects of alternative splicing at the protein functional level have been significantly overestimated [55], others also revealed that the genetic effects on RNA splicing, or sQTLs, are likely primary mediators on complex diseases at many GWAS loci [56–58]. Not only such effects on RNA splicing tend to be highly brain region specific in neurodegenerative disorders [58], but they are largely independent from the effects on RNA expression [56], providing additional links between disease-associated variants and candidate disease genes [48].

### Deconvolution of cell type-specific functional annotations from bulk data

**Figure 2.**
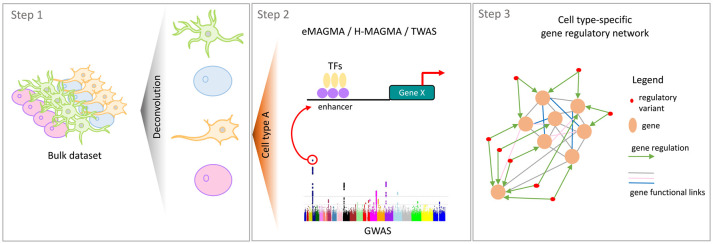
Pipeline for the identification of cell type-specific regulatory processes involved in disease. Cell type-specific functional omics data can now be deconvolved from bulk datasets (Step 1). This can allow to collect transcriptomic and epigenomic data across a larger number of individuals for the discovery of gene regulatory variants. GWAS variants can then be investigated for their functional role on gene expression regulation. This will also help prioritise new causal disease-associated genes (Step 2). Integrative network-based approaches, that build variant and gene relationships, are better suited to study the cumulative effects of disease-associated variants and genes in the dysregulation of sub-networks, or cellular processes (Step 3).

Despite the relevance of the above-mentioned datasets to study human complex diseases at cell type resolution, still the high cost of single cell experiments to study genetic variability across many individuals has prevented so far to build comprehensive cellular gene regulatory maps that would better pinpoint cell type-specific disease-associated genes and cellular processes. This is particularly true for the characterisation of brain regions that are affected in PD, contrary to those affected in AD, such as the cortex and the hippocampus, that have received much more attention in recent years. As a cost-effective solution to such lack of data, new computational tools have been developed to fully exploit informative and publicly available datasets. For instance, projects such as the Religious Orders Study and Memory and Aging Project (ROSMAP) [59,60], the Mayo Clinic Brain Bank [61,62] and the Genotype-Tissue Expression (GTEx) [41] have generated large datasets, including, overall, RNA-seq and matched genotypes from 3387 post-mortem brain samples from multiple brain regions and across 1127 individuals, that have been recently re-examined to extract cell type-specific information [63]. It is, indeed, now possible to characterise the heterogeneity of bulk RNA-seq samples by deconvolving their cellular composition, i.e. the proportion of each cell type, by assuming that bulk RNA-seq data should match the sum of the same set of scRNA-seq data across the different cell types (Figure 2, Step 1). Popular methods are CIBERSORT [64], MuSiC [65], SDCD [66] and SPLITR [63] that have been extensively evaluated [67], and proven effective in predicting from bulk datasets cell type-specific methylation levels [68,69], cell type-specific ATACseq and HiChIP [70] and cell type eQTLs [71,72]. Of note, such methods require evaluation on the combined impact of data transformation, scaling/normalisation, marker selection, cell type composition and choice of methodology on the deconvolution results [66].

A recent study showed that large fractions (81%) of cell type-specific eGenes (genes regulated by eQTLs) identified through deconvolution approaches act in a single cell type and that deconvolved cell-type proportions are highly associated with increased risk for diverse phenotypes/complex traits and with both sex and age of the analysed individuals [63], confirming the necessity to further explore such datasets for a better understanding of disease mechanisms and, possibly, of patient stratification. For instance, substantia nigra-specific datasets exist, such as GTEx for bulk gene expression and the Roadmap project for histone modification marks, that can be specifically used to deconvolve cell type signals for PD research. Furthermore, PPMI RNA-sequencing project [73] includes longitudinal whole blood sequencing data from 1570 subjects. As the transcriptome changes with age and health status, the above mentioned analyses can reveal gene expression changes and pathways disrupted by the disease processes at cell type resolution, also providing the potential to uncover valuable transcriptomic biomarkers.

### Identification of cell type-specific disease-associated genes

The cumulative and combined effects of multiple disease-associated variants on genes can then be investigated through novel computational tools that take into account the contribution of long-range (>10 kb) regulatory interactions (Figure 2, Step 2). For instance, eMAGMA can integrate eQTL data [74], whereas H-MAGMA can leverage signals from subthreshold GWAS loci and can incorporate long-range interactions measured by Hi-C [75]. As shown in [75], H-MAGMA-derived genes can explain a significant proportion of heritability in addition to eQTL-derived genes suggesting that chromatin architecture can provide complementary regulatory phenotypes and that leveraging multiple genomic resources is, therefore, critical for annotating and interpreting GWAS variants. Another useful approach to identify disease variants with cumulative effect on molecular phenotypes is to test association between complex traits and gene expression or alternative splicing through transcriptome wide association analysis (TWAS). Some of the most popular methods are PrediXcan [76], TWAS-Fusion [77,78], and SMR [79] that impute the genotype–expression relationship based on the eQTL association statistics and derive expression–trait associations by correlating the imputed gene expression to the trait, also allowing prediction of whether the disease-associated variants down-regulate or up-regulate the affected genes [75]. A recent study identified both previously known and novel genes whose imputed gene expression and intronic excision levels were significantly associated with AD status helping to pinpoint the likely gene or target of the known susceptibility variants in each disease-associated locus [80]. Similarly, methylome-wide association studies (MWAS) or epigenome-wide association studies (EWAS), that test causal relationships between complex traits and regulatory mechanisms, have proven helpful to complement previous findings and to suggest that the functional effect of disease genetic risk is mediated and enhanced also by the methylation status [81]. A recent study discovered new candidate disease genes whose change in expression, splicing, or methylation are associated with risk of PD with cell type specificity [82].

### Network-based approaches for data integration and identification of dysregulated pathways

Traditional pathway analysis tools can then be used to further characterise the identified disease-associated genes and to test their functional enrichment in cellular processes. Alternatively, network-based approaches can be employed to intersect multiple functional resources and, in turn, to infer functional relationships between genes [83,84]. These methods have been effectively used in the past to identify gene co-regulation patterns for discovery of novel pathways and gene targets in various complex human diseases [85–88]. By map­ping genetic variants onto the interactions between genes, whose functional effects depend on their relationships in the network, these methods can also provide functional and cellular context for disease-associated genetic variants [89] (Figure 2, Step 3). At the molecular level, several types of interactions can be used to define gene relationships. For instance, the strength of gene–gene correlations can be measured by simple gene expression values as well as by other gene expression regulatory mechanisms such as microRNAs, chromosome conformation, epigenetics, and by cell type gene specificity and cell type proportions. Protein–protein interaction resources such as STRING [90], HumanNet [91], and PCNET [92] provide a further source of high-confidence gene functional links that can be filtered based on distinct cell type-specific expressed genes resulting in cell type-specific networks. SCINET [93] and DifferentialNet [94] are examples of recent computational frameworks that allow optimal filtering of a reference network. Genetic interactions or epistasis measure, instead, the synergistic effects of interactions between genetic variants that are related to phenotypes [95] or disease status [96]. For most diseases, genetic interactions between loci might help explain the ‘missing heritability' problem, i.e. the gap between the disease risk explained by the discovered loci and the estimated total heritable disease risk based on familial aggregation [97,98]. However, due to high burden associated with multiple testing and the large sample size necessary to detect genetic interactions, systematic discovery of statistically significant genetic interactions on a genome-scale remains a major challenge. A solution is to restrict the set of variants to be tested through knowledge or data-driven prioritisation [99], for instance, by focusing on the above-mentioned functional regulatory variants, i.e. eQTLs and sQTLs. Interactions between different functional genomic elements are indeed important for understanding regulatory networks. By analysing co-accessibility of different genomic regions, methods such as Cicero [100] enable to detect interactions between transcription starting sites (TSS) and enhancers [101,102], promoters [100], and other genomic elements that can, in turn, provide causal links between gene expression changes. Other methods able to infer directed networks are SCODE [103] and GENIE3[104] that, through pseudotime trajectory analyses of single-cell transcriptomes that order cells along disease progression axes [33], can mimic RNA-seq time series experiments at cell type resolution [105]. It is worth to mention that such approaches also suffer from limitations; protein–protein interaction networks are associated with technical biases inherent to the experimental techniques that can generate false protein links and study biases driven by the research interests [106]. Interconnectedness in gene co-expression networks can be affected by confounding bias, for example generated by different normalisation methods or by the large amount of zero values in single cell RNA-seq data, potentially resulting in false positive findings [107]. A proposed solution is to infer functional associations between genes from the integration of diverse data types and assess them with a novel phenotype-based method [83].

In conclusion, integrative multimodal analysis can better identify diverse variant-to-variant, variant-to-gene and gene-to-gene relationships. In the context of disease, clusters of connected genes can be a proxy for alterations in gene expression regulatory mechanisms [105]. Therefore, identification of functional sub-networks that are characteristic of the disease states may help understand patho­genesis at cell type- and pathway-specific levels and also facilitate identification of key regulators of cellular pathways involved in disease progression which are often potential therapeutic targets and biomarkers for diagnosis.

## Perspectives

- Given the well-recognised cellular heterogeneity of the brain, pinpointing cell type-specific disease variants and their implicated genes is crucial to further understand pathogenicity.
- Since the regulatory program specific to each cell type is the core element governing the cellular identity, cell type-specific multi-layered gene regulatory networks are key tools for the study of disease heritability at cell type level [52].
- Data stratification by sex, age and other phenotypes will provide multiple layers of information that will further benefit our understanding of disease mechanisms [105].

## Open Access Statement

Open access for this article was enabled by the participation of Cardiff University in an all-inclusive Read & Publish pilot with Portland Press and the Biochemical Society under a transformative agreement with JISC.

## Abbreviations

AD: Alzheimer's disease
ER: endoplasmic reticulum
GTEx: genotype-tissue expression
GWAS: genome-wide association studies
ODCs: oligodendrocytes
PD: Parkinson's disease
SNpc: substantia nigra pars compacta
TWAS: transcriptome wide association analysis
